# A bidirectional Mendelian randomization study about the role of morning plasma cortisol in attention deficit hyperactivity disorder

**DOI:** 10.3389/fpsyt.2023.1148759

**Published:** 2023-06-14

**Authors:** Hu Jue, Li Fang-fang, Chen Dan-fei, Chen Nuo, Ye Chun-lu, Yu Ke-pin, Chen Jian, Xuan Xiao-bo

**Affiliations:** ^1^First Clinical School, Zhejiang Chinese Medical University, Hangzhou, China; ^2^Zhejiang Provincial Hospital of Traditional Chinese Medicine, Hangzhou, China

**Keywords:** cortisol, Mendelian randomization, attention deficit hyperactivity disorder, hypothalamic-pituitary-adrenal, single-nucleotide polymorphism

## Abstract

**Context:**

Cortisol, a hormone regulated by the hypothalamic-pituitary-adrenal (HPA) axis, has been linked to attention deficit hyperactivity disorder (ADHD). The nature of the relationship between cortisol and ADHD, and whether it is causal or explained by reverse causality, remains a matter of debate.

**Objective:**

This study aims to evaluate the bidirectional causal relationship between morning plasma cortisol levels and ADHD.

**Methods:**

This study used a bidirectional 2-sample Mendelian randomization (MR) design to analyze the association between morning plasma cortisol levels and ADHD using genetic information from the authoritative Psychiatric Genomics Collaboration (PGC) database (*n* = 55,347) and the ADHD Working Group of the CORtisol NETwork (CORNET) Consortium (*n* = 12,597). MR analyses were employed: inverse variance weighting (IVW), MR-Egger regression, and weighted medians. OR values and 95% CI were used to evaluate whether there was a causal association between morning plasma cortisol levels on ADHD and ADHD on morning plasma cortisol levels. The Egger-intercept method was employed to test for level pleiotropy. Sensitivity analysis was performed using the “leave-one-out” method, MR pleiotropy residual sum, and MR pleiotropy residual sum and outlier (MR-PRESSO).

**Results:**

Findings from bidirectional MR demonstrated that lower morning plasma cortisol levels were associated with ADHD (ADHD-cortisol OR = 0.857; 95% CI, 0.755–0.974; *P* = 0.018), suggesting there is a reverse causal relationship between cortisol and ADHD. However, morning plasma cortisol levels were not found to have a causal effect on the risk of ADHD (OR = 1.006; 95% CI, 0.909–1.113; *P* = 0.907), despite the lack of genetic evidence. The MR-Egger method revealed intercepts close to zero, indicating that the selected instrumental variables had no horizontal multiplicity. The “leave-one-out” sensitivity analysis revealed stable results, with no instrumental variables significantly affecting the results. Heterogeneity tests were insignificant, and MR-PRESSO did not detect any significant outliers. The selected single-nucleotide polymorphisms (SNPs) *F* were all >10, indicating no weak instrumental variables. Thus, the overall MR analysis results were reliable.

**Conclusion:**

The study findings suggest a reverse causal relationship between morning plasma cortisol levels and ADHD, with low cortisol levels associated with ADHD. No genetic evidence was found to support a causal relationship between morning plasma cortisol levels and the risk of ADHD. These results suggest that ADHD may lead to a significant reduction in morning plasma cortisol secretion.

## Introduction

Stress induces a broad range of autonomic, endocrine, and behavioral responses. The hypothalamic-pituitary-adrenal (HPA) axis is a crucial pathway that modulates the stress response by temporarily increasing circulating cortisol levels through the actions of two ligand-activated transcription factors with similar functions: the mineralocorticoid and glucocorticoid receptors. Genetic differences in these receptors may affect the HPA axis’s reactivity, and persistent HPA axis dysfunction has been linked to the emergence of ADHD (1, 2). Early life stress is strongly associated with ADHD-like symptoms, which are thought to involve the growth, development, and differentiation of hippocampal nerves, a key regulatory center of the HPA axis (3). Evidence from a systematic review with meta-analysis indicates a correlation between ADHD associated with lower cortisol levels (4). Compared with normal children of the same age, those with ADHD have lower HPA axis reactivity following stress, which negatively correlates with their symptoms and is also evident in intelligence tests (5, 6). Furthermore, hair dehydroepiandrosterone (DHEA) levels and DHEA/cortisol ratio were independently associated with a composite score of distraction and impulsivity on the Visual-Auditory Continuous Performance of Attention Test (IVA-CPT) (7). Kaneko et al. found that over half of the 30 children with ADHD in their study displayed circadian cortisol secretion disorder, with higher secretion in the early morning and lower secretion at 8 am (8). However, another study showed that ADHD symptoms were strongly correlated with higher levels of cumulative diurnal cortisol, morning cortisol, and afternoon cortisol, even after adjusting for Crohn’s disease (CD), anxiety, and depression symptoms (9). Corominas et al. conducted a comprehensive review of studies on cortisol’s role in ADHD. They found two opposing trends: reduced cortisol responses to stress were linked to comorbid disruptive behavior disorder (DBD), whereas high cortisol responses were associated with comorbid anxiety disorders (AxD) (10). Subjective stress is commonly observed in the clinical environment of individuals with ADHD (11) due to the high prevalence of anxiety and depression symptoms in children and adults (12, 13). Patients’ difficulties in managing routine day-to-day activities owing to the characteristic executive function deficits of ADHD may exacerbate feelings of worry and sadness.

The research mentioned above provides evidence suggesting that ADHD may lead to dysregulation of the HPA axis, potentially resulting in abnormal cortisol levels. However, a recent prospective cohort study involving 126 children aged 4 to 8 has yielded intriguing findings (14). After accounting for variables such as gender, family education background, and personality, the study reveals a correlation between lower levels of hair cortisol in preschool-age children and an increased likelihood of developing ADHD during their school years (14). This observation raises the possibility that low cortisol levels could serve as an early marker for the development and onset of ADHD. Additional insight is provided by a retrospective study conducted by Susan Schloß et al., which suggests that low cortisol levels may indicate an escalation in ADHD symptoms among 4–5-year-old children (15). Notably, this correlation appears more prominent in boys, suggesting a potential gender-related aspect to the association between low cortisol levels and early ADHD. Nevertheless, it is important to note that the study’s retrospective nature limits causal interpretations, and the observed associations can only establish correlation. In addition, cortisol levels are influenced by various biological and environmental factors, necessitating further research to validate these findings and gain a comprehensive understanding of their potential diagnostic or intervention applications for ADHD. So it remains to be clarified whether ADHD leads to the disruption of cortisol levels or disrupted cortisol levels lead to ADHD.

However, a cross-sectional descriptive study from Asia found no significant difference in serum cortisol levels between ADHD patients and controls (16). They may be impacted by various factors, including the impact of co-morbidities, characteristics of the study participants, such as medication use, presence of other conditions, and age, as well as the techniques used to measure cortisol levels, such as using saliva or plasma samples. Despite these challenges, research shows that HPA axis dysfunction in individuals with ADHD strongly correlates with the primary symptoms of hyperactivity, impulsivity, and inattention. These findings prompted further investigation into the role of morning cortisol secretion in the complex interplay between cortisol and ADHD.

Twin studies have shown that ADHD has a heritability rate of 70–80% throughout an individual’s lifetime, based on the consensus of over 30 studies (17, 18). A genome-wide meta-analysis of ADHD has established that a clinical diagnosis of ADHD is made based on the excessive manifestation of one or more heritable quantitative traits (19). To overcome the limitations of observational studies, a technique called MR can establish causality between an exposure and a consequence. MR utilizes genetic variations randomly assigned at conception as a proxy for an exposure that occurs before the onset of the disease (20), which helps overcome the issue of reverse causation. This study used a bidirectional, two-sample MR approach to investigate the relationship between genetically determined morning plasma cortisol levels and ADHD and genetically determined ADHD and morning plasma cortisol levels.

## Materials and methods

**FIGURE 1:**
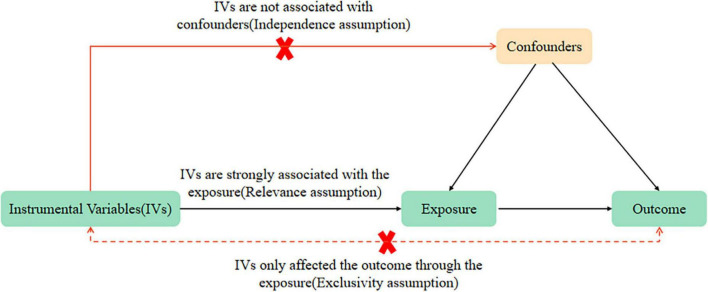
Mendelian randomization (MR) analysis’s three key assumptions: relevance assumption, independence assumption, and exclusivity assumption.

Mendelian randomization analysis relies on three key assumptions (21): (i) a connection between genetic variations and the exposure of interest (relevance assumption), (ii) genetic differences are independent of confounding factors in the relationship between the risk factor and outcome (the assumption of independence), and (iii) the influence of genetic variations on the exposure primarily determines the outcomes (exclusivity assumption). In Mendelian MR analysis, genetic markers such as SNPs are employed as substitutes for the exposure of interest to control for confounding factors that may distort the results of observational studies. SNP is a substitution of a single nucleotide that occurs at a specific position in the genome, where each variation is present at a level of more than 1% in the population. SNPs underline differences in our susceptibility to a wide range of diseases. This study utilized data sets from the CORtisol NETwork (CORNET) and ADHD in the PGC ADHD database for the MR analysis. In this MR analysis, all three hypotheses were met by the selected instrumental variables. Firstly, we included SNPs significantly associated with morning plasma cortisol levels through the CORNET Consortium. Secondly, we selected strong instrumental variables with *F* > 10. Thirdly, we excluded SNPs with linkage disequilibrium effects, fulfilling hypothesis i. Additionally, to include SNPs that were only associated with the outcome through exposure, with genetic variation unrelated to any confounders affecting ADHD and unrelated to confounders along the exposure-outcome pathway, we screened phenotypes significantly associated with SNPs through the PhenoScanner database and determined whether these phenotypes were confounders of ADHD by previously published MR studies. If yes, the involved SNPs were removed to satisfy hypotheses ii and iii. The same approach was used in turn (Figure 1).

### Two-sample MR design

To address these questions, we employed a two-sample MR design in our study, in which two types of data are obtained from two non-overlapping datasets for MR analysis (22). This approach was chosen due to the inherent difficulty in obtaining comprehensive data encompassing genetic variation, exposure, and the relationship between genetic variation and outcomes within the same sample. Adopting a two-sample MR design gives us several advantages over a single-sample design. Firstly, weak instrument bias in a two-sample design tends to favor the null hypothesis, making it more conservative and reducing the likelihood of false positive results compared to bias in the direction of correlation. Secondly, in scenarios where it is challenging to simultaneously measure exposure and outcome data within the same group of individuals, the two-sample MR approach significantly expands the scope of MR studies (23). According to previous research, the heritability of morning plasma cortisol ranges from 30 to 60% (24, 25). Identifying genetic variations that contribute to changes in morning cortisol values could provide critical insight into the mechanisms of HPA axis activation (26). The CORNET consortium aims to identify genetic determinants of inter-individual variation in HPA axis function. For example, the consortium has discovered two genes, SERPINA6 and SERPINA1, highly expressed in tissues involved in cortisol physiology. SERPINA6 encodes corticosteroid-binding globulin, a protein that binds cortisol in the bloodstream, while SERPINA1 encodes α1-antitrypsin, a protein that regulates the release of cortisol from corticosteroid-binding globulin by inhibiting the cleavage of the reactive center loop (27). In the forward direction, the CORNET consortium provided the GWAS associated with the levels of serum morning plasma cortisol as the sample exposure, which was obtained from a sample of 12,597 individuals of European ancestry (on average 53.5 years old, 59.2% female) and replicated in an independent sample of 2,795 participants. Morning plasma cortisol levels were standardized using the plasma cortisol log-scale SD score corrected for genetic control genomic control and adjusted for age and sex (27). SNPs were identified in or in proximity to specific genes. The GWAS summary statistics from the Psychiatric Genomics Consortium (PGC) ADHD database, including 35,191 controls from 12 cohorts (19) and 20,183 patients with ADHD, were used as sample outcomes. Throughout our study, we considered effect sizes based on the European participants (19,099 cases and 34,194 controls) to establish the relationship between morning plasma cortisol levels and ADHD. In the reverse direction, we used the GWAS summary statistics from the ADHD Working Group of the PGC database (*n* = 55,347) as the discovery sample exposure and cortisol obtained from the CORNET consortium (*n* = 12,597) as the sample outcome to investigate the causal relationship of ADHD on morning plasma cortisol levels.

### Instrumental variable selection

The CORNET consortium identified SNPs that were highly (*P* < 5 × 10^–6^) and independently (*r*^2^ < 0.01, window size = 10000 kb) associated with morning plasma cortisol levels. Typically, SNPs with *P* < 5 × 10^—8^ are considered significant across the entire genome. However, only one SNP was screened at this threshold value, so the threshold was appropriately adjusted downward. Therefore, the selected instrumental variables meet the correlation assumption, i.e., there is a strong association between the exposure factors and instrumental variables. The Two-Sample MR R software package was used to exclude SNPs with disequilibrium in the links through the clump step. The parameters used were *r*^2^ = 0.01 and kb = 10000, indicating that SNPs with *r*^2^ greater than 0.01 within a 10 MB range, including the most important SNPs, were removed to eliminate the effect of linkage disequilibrium (LD). To verify whether the selected instrumental variables met the independence assumption, Pheno Scanner^1^ was used to examine the association of the remaining SNPs with other phenotypes. The results showed that the remaining five SNPs were not associated with prenatal exposure to alcohol, tobacco, cocaine, prematurity, or other factors that may affect ADHD, confirming the independence hypothesis. Finally, the five extracted SNPs were matched with SNPs from the PGC ADHD database, and the corresponding data were collated and merged. We extracted the cortisol-associated SNPs for ADHD from a GWAS analysis in the PGC ADHD database, and we considered impact estimates from European participants, including 19,099 cases and 34,194 controls. SNPs that demonstrated a robust association with the statistical threshold for the GWAS (*P* < 5 × 10^–8^, linkage disequilibrium *r*^2^ = 0.01, window size = 10000 kb) were selected as instrumental genetic variables. The CORNET consortium (27) then collected genetic correlations among those with cortisol with European ancestry. In the end, Pheno Scanner was used to verify that the selected instrumental variables met the independence assumption (28), and seven SNPs were retrieved and matched with SNPs from the CORNET consortium.

### Statistical analysis

In this study, we assessed the influence of a specific genetic variation of SNPs on exposure by determining its effect on an outcome. We then performed MR analyses to combine the estimates obtained using IVW, MR-Egger regression, Weighted median, Simple mode, and Weighted mode. The IVW method was predominantly used for analysis. The analysis outcome was presented as odds ratios (ORs) and a 95% confidence interval (CI).

### Sensitivity analyses

We utilized various reliable methods to verify and correct the estimates obtained, including weighted median, MR-Egger regression, and MR-PRESSO (29–31). It has been demonstrated that combining the weighted median method with the MR-Egger regression method can help identify horizontal pleiotropy and provide an unbiased estimate of the causal influence. We used the weighted median approach to compare the two methods, allowing up to 50% of incorrect or pleiotropic instrumental factors in the analysis. MR studies (32, 33) require that instrumental variables can only influence outcomes through the studied exposure factor and are not directly related to outcomes. Testing the exclusivity hypothesis is challenging as genetic variants are all pleiotropic. The intercept term of MR–Egger regression is now commonly used to test for the presence of pleiotropy. When the linear regression intercept Egger–intercept of the MR–Egger model is close to 0 (*P* > 0.05), it indicates no pleiotropy in the instrumental variables, and the exclusion hypothesis can be considered valid; conversely, it indicates the presence of genetic pleiotropy and the exclusion hypothesis is invalid. In addition, the MR-PRESSO method was used to identify and correct outliers in the IVW linear regression. It consists of three components: (a) pleiotropy detection (MR-PRESSO global test), (b) pleiotropy correction by removing outliers (MR-PRESSO outlier test), and (c) study of statistically significant variations between the causal estimates before and after outlier removal (MR-PRESSO distortion test). We presented (31) the causative estimates for associations that have been corrected for outliers if the *P*-values of the global and distortion tests indicated a probability smaller than 0.05, suggesting the presence of horizontal pleiotropy. The Cochran *Q* statistic and *P*-value (34) were utilized to examine heterogeneity in the IVW estimations to corroborate the findings further. In addition, the results were analyzed separately using the leave-one-out method for sensitivity analysis. The leave-one-out method is a widely used technique for sensitivity analysis, whereby each SNP is removed one by one, and the results are observed to be statistically different before and after the removal. If *P* > 0.05 is obtained after excluding an SNP, the SNP does not have a non-specific effect on the effect estimation results (35). All analyses were conducted using R version 4.1.1 (Statistical Computing with R Foundation), and the R packages “Two Sample MR” (35) and “MR-PRESSO” (31) were used for MR analysis.

### Assessment of assumptions

The statistical validity of SNPs and MR studies is crucial for the unbiased causal estimation of MR. The statistical validity of SNPs depends on the explanatory power of SNPs for the phenotype (*R*^2^), which can be assessed by the strength of the correlation between SNPs and the phenotype (*F*-statistic). The magnitude of *R*^2^ ranges from 0 to 1, with a larger *R*^2^ indicating a greater explanation of the phenotype by SNPs. The calculation of *R*^2^ in this study was mainly based on the work of Papadimitriou et al. (36), published in Nature Communications. When the number of SNPs was less than or equal to 10, *R*^2^ was calculated using the following formula: *R*^2^ = 2 × *EAF* × (1-*EAF*) × *beta*^2^. When the number of SNPs exceeds 10, the following equation was used to calculate it: *R*^2^ = 2 × *EAF* × (1-*EAF*) × *beta*^2^[2 × *EAF* × (1-*EAF*) × *beta*^2^ + 2 × *EAF* × (1-*EAF*) × N × se (*beta*) ^2^]. The *F*-statistic reflects the strength of the correlation between SNPs and phenotypes and is commonly used to determine whether SNPs are weak instrumental variables (37). A smaller *F*-statistic generally indicates a greater likelihood of bias in MR findings. In this study, the *F*-statistic was assessed using the following equation: *F* = (N-K-1)/K × [*R*^2/^(1-*R*^2^)], where N is the sample size, K is the number of SNPs, and *R*^2^ is the degree of explanation of exposure by SNPs. The size of the *F*-statistic decreases as the number of SNPs increases and increases as the sample size and the degree of explanation of exposure by SNPs increase. An *F* > 10 is considered a strong instrumental variable, while an *F* < 10 is weak. The *F* statistic corresponding to each SNP was calculated in this study (Tables 1, 3). Its distributions ranged from 22.57 to 52.72, with *F*-values > 10 indicating that the results are not weakly biased by IVs and are reliable.

**TABLE 1:** The genome-wide significance of certain SNPs in relation to the natural log-transformed morning plasma cortisol levels and the correlation between these SNPs and ADHD.

SNP					Plasma cortisol					ADHD				Palindromic +ambiguous
		Eaf	*R* ^2^	*F*	A1	A2	Beta	SE	*P*-value	A1	A2	SE	*P*-value	
rs1075533	RP11-563P16.1	0.037	0.002	24.45	A	G	−0.17158	0.0347	7.740e-07	A	G	0.0302	0.5883	False
rs12589136	SERPINA6	0.217	0.004	48.81	T	G	0.10340	0.0148	3.320e-12	T	G	0.0159	0.6756	False
rs1340395	RP11-202K23.1	0.926	0.002	23.80	T	C	−0.12635	0.0259	1.090e-06	T	C	0.0253	0.6687	False
rs4400057	DPP3P1	0.912	0.016	24.06	A	G	−0.31933	0.0651	9.460e-07	A	G	0.0200	0.6623	False
rs6830	DNAL1	0.320	0.002	22.57	A	G	−0.06318	0.0133	1.940e-06	A	G	0.0141	0.8234	False

### Ethics approval

No ethics approval was needed because all analyses in the present investigation used only publicly accessible data.

## Results

### MR analysis of the causal relationship between morning plasma cortisol levels on ADHD

**FIGURE 2:**
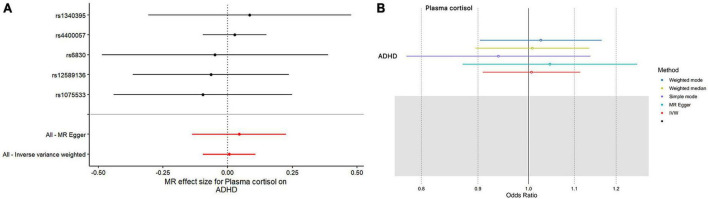
The outcomes of single- and multi-SNP analyses investigating the impact of SNPs on natural-log transformed morning plasma cortisol levels in relation to ADHD. **(A)** The black lines show the findings of single-SNP analyses, while the red shows the results of multi-SNP analyses. **(B)** Applying the IVW, MR-Egger, weighted median, simple mode, and weighted mode for MR analysis.

**FIGURE 3:**
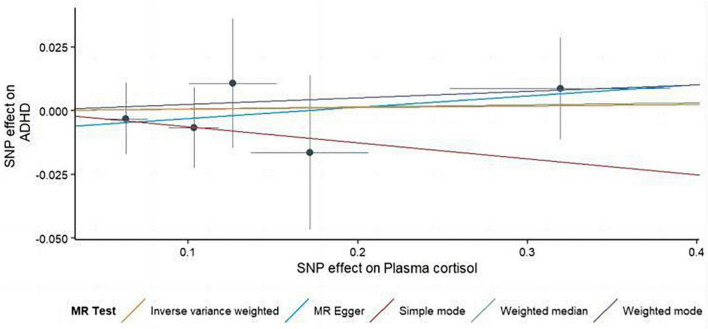
Using several MR techniques, scatterplots demonstrate genetic linkages between natural logarithm-transformed morning plasma cortisol levels and the risk for ADHD. The inclinations of the lines in the plots demonstrate the causal relationship for each methodology employed.

This study used MR analysis to evaluate the potential causal relationship between morning plasma cortisol levels on ADHD. Information on the five SNPs that achieved genome-wide significance (*P* < 5 × 10^–6^) for morning plasma cortisol levels is presented in Table 1. The *R*^2^ was 2.6%, and the distribution of the *F*-statistic corresponding to each SNP ranged from 22.57 to 48.81, while the *F*-statistic corresponding to all five SNPs was 67.28. These findings suggest that weak instrumental variable bias was less likely to impact the causal association. We observed no evidence of pleiotropy (MR-Egger Intercept: −0.008, SE = 0.0151; *P* = 0.653) or heterogeneity [Cochran’s *Q* (df = 3) = 0.879, *P* = 0.928], and MR-PRESSO did not detect any outliers. The aggregate estimations obtained by IVW or MR-Egger did not indicate any cortisol-related risk of ADHD (Table 2, Figures 2, 3 and Supplementary Figures 1, 2). Sensitivity analyses utilizing the weighted median, simple mode, and weighted mode did not reveal any significant associations (Table 2 and Figure 3), and MR-PRESSO failed to detect any significant link between cortisol and ADHD (β = 0.006, SD = 0.024, *P* = 0.815). Moreover, the leave-one-out sensitivity analysis of the IVW method demonstrated that even after gradually removing all five SNPs, the remaining four SNPs showed similar results to the combined effect OR of the IVW method, with *P*-values greater than 0.05. No SNPs with strong effects on the results were found in IV, indicating that the effect OR of the previous IVW method was robust (Supplementary Figure 2).

**TABLE 2:** The OR values of IVW, MR-Egger, weighted median, simple mode, and weighted mode and their 95% CIs.

Exposure	Outcome	Method	SE	Beta	OR (95% CI)	*P*	IVW		MR-Egger	
							*Q*	*P*	Intercept	*P*
Plasma cortisol	ADHD	MR-Egger	0.093	0.044	1.045 (0.872–1.252)	0.665			−0.008	0.653
		Weighted median	0.060	0.008	1.008 (0.895–1.136)	0.897				
		IVW	0.052	0.006	1.006 (0.909–1.113)	0.907	0.879	0.928		
		Simple mode	0.098	−0.063	0.939 (0.781–1.129)	0.540				
		Weighted mode	0.065	0.025	1.026 (0.905–1.163)	0.712				

**TABLE 3:** Morning plasma morning levels and genome-wide significant SNPs for naturally log-transformed ADHD.

SNP		Eaf	Chr	Position	*R* ^2^	*F*	ADHD					Plasma cortisol					Palindromic+ ambiguous
							A1	A2	Beta	SE	*P*-value	A1	A2	Beta	SE	*P*-value	
rs10262192	FOXP2	0.4322	7	114091753	0.003	30.752	A	G	0.07320	0.0132	2.887e-08	A	G	−0.004	0.0127	0.7447	False
rs112984125	ST3GAL3	0.3120	1	44237465	0.005	52.722	A	G	−0.10601	0.0146	3.581e-13	A	G	0.018	0.0134	0.1826	False
rs1427829	RP11-1109F11.3	0.5575	12	89760744	0.003	36.090	G	A	−0.07990	0.0133	1.822e-09	G	A	0.015	0.0132	0.2508	False
rs212178	LINC01572	0.9004	16	72653326	0.003	33.293	A	G	−0.11540	0.0200	7.677e-09	A	G	0.025	0.0217	0.2428	False
rs4858241	SGO1-AS1	0.3620	3	20669071	0.002	31.761	G	T	−0.07890	0.0140	1.740e-08	G	T	−0.029	0.0340	0.3880	False
rs4916723	LINC00461	0.4397	5	87854395	0.003	32.195	C	A	0.07660	0.0135	1.576e-08	C	A	0.0005	0.0129	0.9662	False
rs9677504	SPAG16	0.1100	2	215181889	0.003	32.203	A	G	0.11690	0.0206	1.391e-08	A	G	−0.044	0.0200	0.0273	False

### MR analysis of the causal relationship between ADHD on morning plasma cortisol levels

**FIGURE 4:**
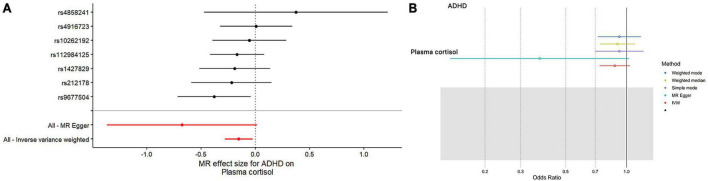
The results of both single- and multi-SNP analyses on the SNP influence of ADHD risk on morning plasma cortisol levels that have undergone a natural log transformation. **(A)** The red lines show the results of a multi-SNP analysis, whereas the black lines show the findings of a single SNP analysis. **(B)** Applying the IVW, MR-Egger, weighted median, simple mode, and weighted mode for MR analysis.

**FIGURE 5:**
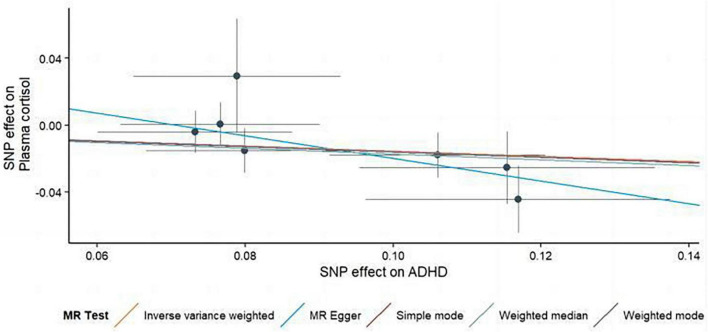
Scatterplots generated through different MR techniques demonstrate the genetic connections between natural-log transformed ADHD and morning plasma cortisol levels risk. The slopes of each line demonstrate the causal connection for each strategy.

In this MR Analysis, we aimed to investigate the causal relationship between ADHD on morning plasma cortisol levels. Table 3 displays data on the associations of the seven selected SNPs with ADHD and morning plasma cortisol, with *P*-values and effect estimates calculated for Europeans. We calculated an *R*^2^ of 2.2%, and the distribution of the *F*-statistic corresponding to a single SNP ranged from 31.76 to 52.72, while the *F*-statistic corresponding to seven SNPs was 173.137. Given that *F* > 10, the likelihood of weak instrumental variables was relatively small, indicating that the results of the MR analysis were unlikely to be affected by weak instrumental variable bias. The MR-Egger regression intercept term was 0.048, indicating no genetic pleiotropy between SNPs and morning plasma cortisol levels (*P* = 0.192). The MR-PRESSO test showed no genetic pleiotropy bias or outliers (β = −0.001, SD = 0.019, *P* = 0.892). Contrary to the previous MR analysis results, our findings revealed a negative causal association between ADHD and morning plasma cortisol levels (IVW method: *b* = −0.154, *P* = 0.018) (Table 4 and Figures 4, 5). Sensitivity analyses using IVW, MR-Egger regression, Weighted median, Simple mode, and Weighted mode were conducted, with results in Table 4 and Figure 5. Leave-one-out and funnel plot analyses indicated no effect (Supplementary Figures 3, 4). The IVW method showed that each 1 SD increase in clinical ADHD risk resulted in a 14.3% decrease in morning plasma cortisol levels (OR = 0.857, 95% CI: 0.755–0.974). Weighted median regression yielded similar results (OR = 0.842, 95% CI: 0.714–0.992). MR Egger’s results revealed no statistically significant association between clinical ADHD risk and morning plasma cortisol levels (OR = 0.509, 95% CI: 0.255–1.015). Simple mode and Weighted mode provided results similar to MR Egger’s results. The direction of the causal effect obtained by combining these five was consistent (Figure 5). Heterogeneity tests and sensitivity analyses of IVW (*P* = 0.594) and MR-Egger regression (*P* = 0.192) with Cochran’s *Q*-test [Cochran’s *Q* (*df* = *6*) = 4.618, *P* = 0.594] indicated no heterogeneity in SNPs (Table 4). When using only one SNP as the independent variable (IV), the funnel plot (Supplementary Figure 3) reveals that the dots reflecting the effect of the causal relationship are symmetrically distributed, indicating that the causal association is less likely to be impacted by potential bias. The “Leave-one-out” sensitivity analysis results (Supplementary Figure 4) showed that the *P*-value range and IVW analysis results of the remaining six SNPs were similar to those of the included SNPs, and no SNPs were found to have a significant effect on the causal association estimates after excluding each SNP in turn.

**TABLE 4:** The OR values of IVW, MR-Egger, weighted median, simple mode, and weighted mode and their 95% CIs.

Exposure	Outcome	Method	SE	Beta	OR (95% CI)	*P*	IVW		MR-Egger	
							Q	*P*	Intercept	*P*
ADHD	Plasma cortisol	MR-Egger	0.519	−0.988	0.509 (0.255–1.015)	0.113			0.048	0.192
		Weighted median	0.101	−0.104	0.842 (0.714–0.992)	0.040				
		IVW	0.087	−0.135	0.857 (0.755–0.974)	0.018	4.618	0.594		
		Simple mode	0.140	−0.082	0.851 (0.678–1.068)	0.213				
		Weighted mode	0.125	−0.082	0.853 (0.697–1.045)	0.176				

## Discussion

This two-way 2-sample MR study provides evidence against a causal relationship between morning plasma cortisol levels and ADHD. However, our findings suggest a causal relationship of ADHD on morning plasma cortisol levels. To establish the direction of this association, we conducted bidirectional MR analysis and implemented several sensitivity analyses using various MR techniques to assess the robustness of our findings. The primary objective of our study was to investigate the bidirectional association between morning plasma cortisol levels and ADHD using a two-sample MR approach. Our results shed light on how ADHD impacts morning plasma cortisol levels. Normally, cortisol levels in the blood follow a diurnal rhythm, rising in the morning, peaking at around 8 am, a sharp decline over the following few hours, and progressively decreasing throughout the day, reaching a nadir around bedtime (38, 39). Cortisol, the end product of the HPA axis, plays a crucial role in individual emotion regulation and behavior control and can increase hormone levels via various pathways, resulting in a stress response, which is an indicator of acute and chronic stress (40). Evidence suggests that chronic stress, particularly in childhood, may contribute to the development of ADHD, which is highly comorbid with anxiety (41). Exposure to adversity and chronic stress in childhood may lead to HPA axis hyporeactivity later in life (42). Morning cortisol levels can partially reflect HPA axis activity. In the first part of our MR analysis, we failed to establish a potential causal relationship between morning cortisol levels and ADHD, using morning cortisol levels as an indicator of exposure and ADHD as an outcome indicator. It may be due to the complex etiology of ADHD, which involves genetic factors, neuroanatomy and neurochemistry, and central nervous injury, with maternal pregnancy stress and family social adversity among the influential factors (43). In the GWAS of morning plasma cortisol levels, the SNP in the SERPINA gene had the highest *F*-value (*F* = 48.81). It has been shown that (27) inter-individual differences in morning plasma cortisol levels in individuals of European ancestry are largely attributable to genetic variation in the region of chromosome 14 containing the SERPINA6 and SERPINA1 genes, suggesting that the SERPINA gene exerts an important influence on plasma cortisol levels and may provide clinical insights for future HPA axis adjustments to reduce ADHD symptoms.

The second part of this study examined the inverse causality of ADHD on morning plasma cortisol levels using MR analysis, which is consistent with the majority of studies, indicating that ADHD has a susceptibility to low morning plasma cortisol levels. Our research found that for every increase in one standard deviation of ADHD, the risk of decreased morning cortisol levels will increase by 14.3% (OR = 0.857, 95% CI: 0.755–0.974, *P* = 0.018). ADHD is a disease of neurodevelopmental disorder that does not correspond to the developmental level (or to age). It manifests itself in various situations in school, family, and social environments, characterized by attention deficit, hyperactivity, and impulsivity (44), and has been associated with low morning plasma cortisol levels in numerous studies. For example, Chinese children with all subtypes of ADHD, as well as individuals with clinical ADHD in England and Germany, have been found to have lower basal cortisol levels than healthy children (14, 45, 46). According to meta-analyses (4), Adolescents with ADHD exhibit lower baseline and morning cortisol levels than TD children. Since their cortisol levels take longer to peak, children and adolescents with ADHD may experience weariness and have a delayed rising time in the morning (47). Children with ADHD may also have an irregular diurnal cycle of cortisol levels, resulting in low morning cortisol levels. Researchers showed that persons with ADHD had a delay of 2 h in the phase of their cortisol cycles (48). Shin and Lee (6) found that lower cortisol levels were correlated with poor test performance in children. As a result, it is probable that some ADHD kids’ inability to achieve optimal neurocognitive function is caused by compromised HPA axis function. ADHD is a complex neurodevelopmental disorder characterized by cognitive impairments and atypical brain circuitry, which exhibit substantial variability across individuals. Some children diagnosed with ADHD manifest reduced activation in the alerting network, comprising frontoparietal and thalamic circuits (49). These deviations in brain function have been associated with low cortical arousal and attention deficits, including difficulties sustaining attention over time. Additionally, prior research has highlighted the potential utility of assessing HPA axis activity as an early indicator of basal attention system hypoactivity or impairment. Specifically, evidence suggests that HPA axis activity may be linked to deviations in frontal brain regions, thereby impeding the proper functioning of this critical alertness network (50). However, it is crucial to acknowledge the clinical complexity of ADHD and tailor diagnostic and treatment strategies to accommodate individual variability in symptom presentation. Two-sample MR and population-based sibling comparison study (51) found that individuals with ADHD are significantly more likely to develop post-traumatic stress disorder (PTSD) later in life due to HPA axis hyporeactivity. In addition, Llorens M’s study (52) has found a relationship between HPA axis hormone levels, the degree of cognitive and inattention symptoms present in individuals with ADHD, and how sex and abuse during childhood affect these symptoms.

The mechanism of HPA axis dysfunction caused by ADHD may be explained as follows: Glucocorticoid (GC), which is a product of the HPA axis, is essential for many behaviors and advanced brain functions of the mammalian central nervous system, such as cognition, emotion, memory, and attention. When GC levels are low, executive behavior abilities are reduced, symptoms related to ADHD appear, and self-control abilities are poor, leading to hyperactivity and difficulty remaining calm (53). The hippocampus regulates various functions, including learning and memory, behavior execution, and endocrine and autonomic nervous activities. GC exerts its extensive biological effects primarily through the glucocorticoid receptors (GR), which are concentrated in the hippocampal CA1 region and dentate gyrus (54). Altered expression of GR can lead to impaired binding with GC, preventing the proper functioning of endogenous GC and ultimately resulting in HPA axis dysfunction. Medications used to treat ADHD, such as methylphenidate, have been shown to increase cortisol levels in patients by promoting dopamine release in the central nervous system (55). After 1 month of methylphenidate hydrochloride treatment, children with ADHD significantly increased cortisol levels, positively correlated with neuropsychological performance over 6 months of treatment (56). Glucocorticoids have been shown to improve impulse control in continuous performance tests (56), potentially by boosting the effects of dopamine in the mesolimbic pathway (57). In addition, stimulant medication may increase baseline cortisol levels (58). Lower cortisol levels have been found to predict HPA axis impairment (59). Our MR analysis study indicated a negative correlation between clinical ADHD and morning plasma cortisol levels, suggesting that ADHD may lead to low morning cortisol levels. However, whether low cortisol levels exacerbate clinical symptoms of ADHD, we have not established a causal relationship in our MR analysis. Further clarification is pending with larger sample sizes and more clinical studies in the future.

### Limitations

Despite using MR analysis to reduce residual confounding bias, there are several limitations to consider in this study. First, one limitation is that a more liberal threshold (*P* < 5 × 10^–6^, *r*^2^ = 0.01) was used to select SNPs associated with morning plasma cortisol levels for analysis since only one SNP reached the genome-wide significance level (*P* < 5 × 10^–8^, *r*^2^ = 0.001) in the initial screening this may have introduced weak instrument bias. This study calculated the explanatory power of SNPs for phenotypes (*R*^2^) and the strength of association between SNPs and phenotypes to assess the statistical validity of SNPs in the MR study (*F*-statistic) (Tables 1, 3). The likelihood of weak instrument bias (60) may be mitigated by the fact that the *F*-statistic of each included SNP was greater than 10. However, even though the *F*-statistic was greater than 10 in all MR studies, the explanatory power of SNPs for cortisol (*R*^2^ = 0.026) and ADHD (*R*^2^ = 0.022) was low; therefore, bias in MR study results cannot be ruled out due to insufficient statistical power of SNPs. Second, it is widely believed that using different databases for different races may affect the results and cause population stratification bias. Population stratification refers to changes in the frequency of genetic variants across populations with various genetic origins, leading to false links between genetic variants and outcomes. Population stratification in MR investigations may lead to unjustified assumptions of independence or exclusivity and, therefore, incorrect causal conclusions. For example, Haworth et al. (61) found that genetic variation and primary health outcomes were associated with place of birth using UK Biobank data; failure to well-correct for population stratification can lead to spurious associations between genetic variation and primary health outcomes. To avoid population stratification bias, the most direct way is to include populations with the same genetic background for genetic association studies. However, due to traditional GWAS’s low statistical test efficacy, there has been a trend to expand the sample size for multicenter GWAS. In this study, the ADHD Working Group of the PGC database comprised a population-based cohort of 14,584 adults with ADHD and 22,492 control subjects. The GWAMA of morning plasma cortisol levels examined 12,597 people of European descent. Nonetheless, the analysis considered effect sizes based on the European members of the Working Group of the PGC database (19,099 cases and 34,194 controls). Only about 3.7% of the 55,347 participants were non-Europeans, so it is believed that such a small percentage of non-Europeans doesn’t affect the accuracy of the results. Third, our analysis was limited to morning plasma cortisol data; therefore, we could not examine the relationship between ADHD risk and cortisol levels throughout the day. It is worth noting that cortisol secretion exhibits a diurnal pattern, and incorporating data on cortisol fluctuations over a day could provide additional information. In addition, it is worth noting that after the submission of this manuscript, the Nature Genetics Journal updated the latest ADHD GWAS to include 27 markers (62). The results of this updated GWAS may refine and complement the conclusions of our study. We plan to conduct further discussions and studies in the future.

## Conclusion

This study represents the first MR investigation to explore the causal relationship between morning plasma cortisol levels and ADHD. This study represents the first application of MR to investigate the causal association between morning plasma cortisol levels and ADHD. The findings from our study provide that ADHD is inversely and causally related to morning plasma cortisol levels. However, no genetic evidence supports that morning plasma cortisol levels are causally associated with ADHD. Our study’s results suggest that ADHD causes reduced morning plasma cortisol levels. In the future, larger and more in-depth cortisol and ADHD GWAS studies are needed to determine how ADHD affects disruption in the cycles of cortisol secretion.

## Data availability statement

The original contributions presented in the study are included in the article/Supplementary material, further inquiries can be directed to the corresponding authors.

## Author contributions

HJ proposed the concept, design of the study, and drafted the main manuscript. CD-f and LF-f executed the collection and analysis of data. CN, YC-l, and YK-p accomplished the literature search. XX-b and CJ responsible for editing and critical revision. All authors have read and agreed to the published version of the manuscript.

## Footnotes

1
http://www.phenoscanner.medschl.cam.ac.uk
