# Supplementary material for: A bidirectional Mendelian randomization study about the role of morning plasma cortisol in attention deficit hyperactivity disorder
Source: Front Psychiatry. 2023 Jun 14;14:1148759. doi: 10.3389/fpsyt.2023.1148759 (PMC10303788; doi:10.3389/fpsyt.2023.1148759)
Supplement: Supplementary file 1 [file Data_Sheet_1.docx]

**Fig. S1** Funnel plot for MR analysis with morning plasma cortisol levels as exposure and risk for ADHD as the outcome


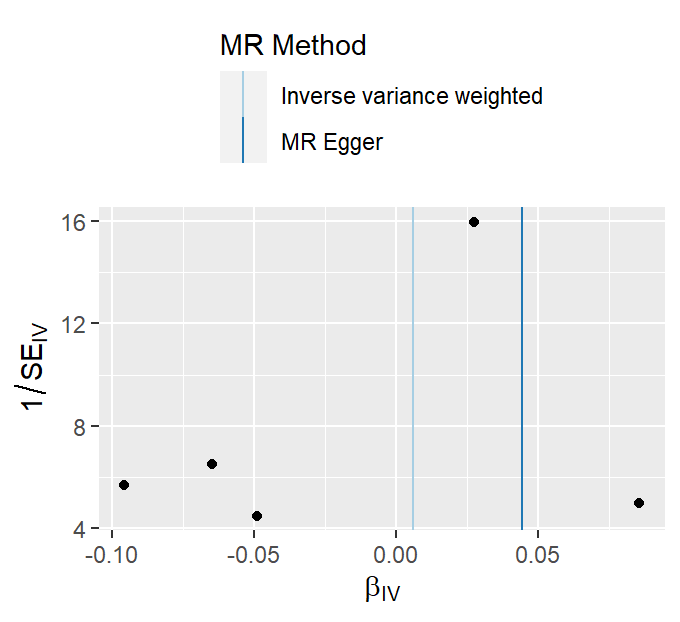


**Fig. S2** The leave-one-out approach used to examine the association between morning plasma cortisol levels as the independent variable and the potential for ADHD as the dependent variable

**
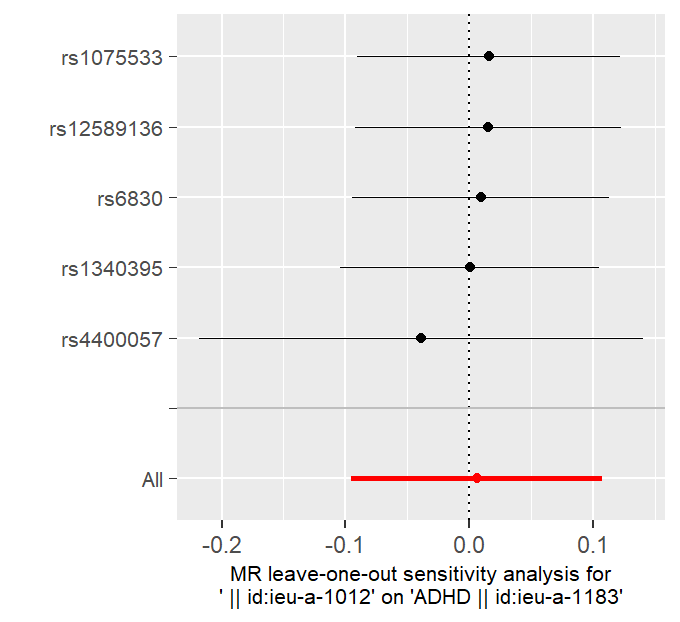
**

**MR leave-one-out sensitivity analysis for ‘Plasma cortisol’ on ‘ADHD’**

**Fig. S3** Funnel plot for MR with the amount of morning plasma cortisol levels as the outcome and the risk for ADHD as the exposure

**
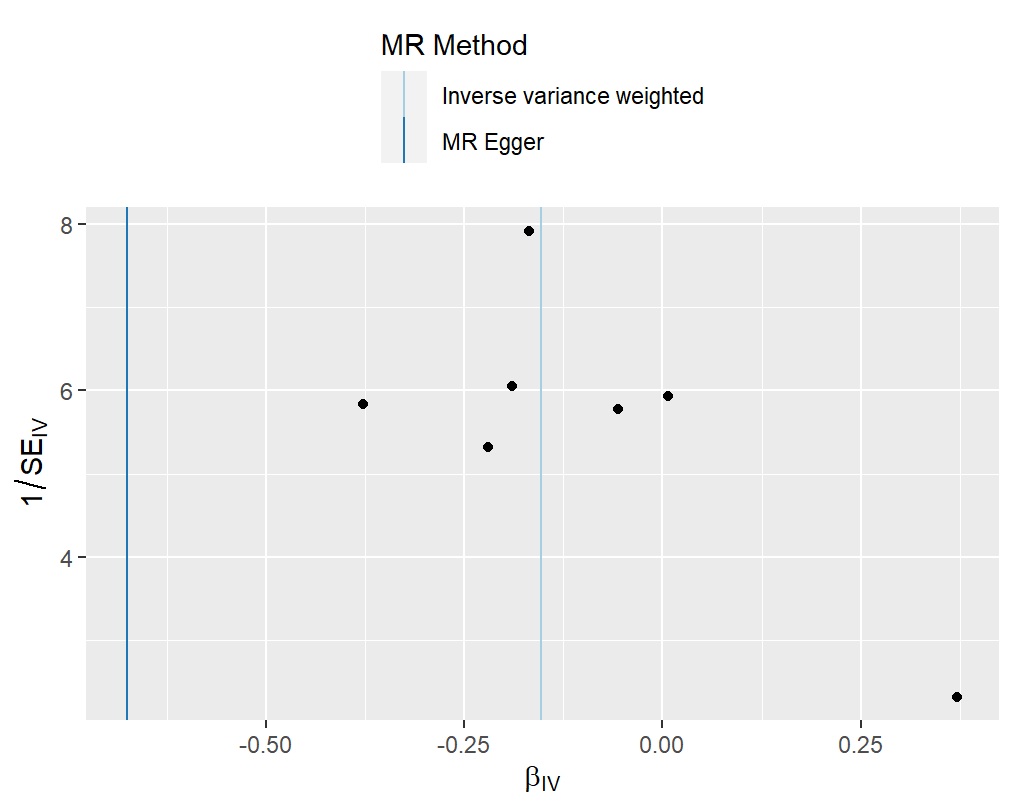
**

**Fig. S4** The leave-one-out approach used to examine the association between ADHD as the independent variable together with the potential for morning plasma cortisol levels as the dependent variable

**
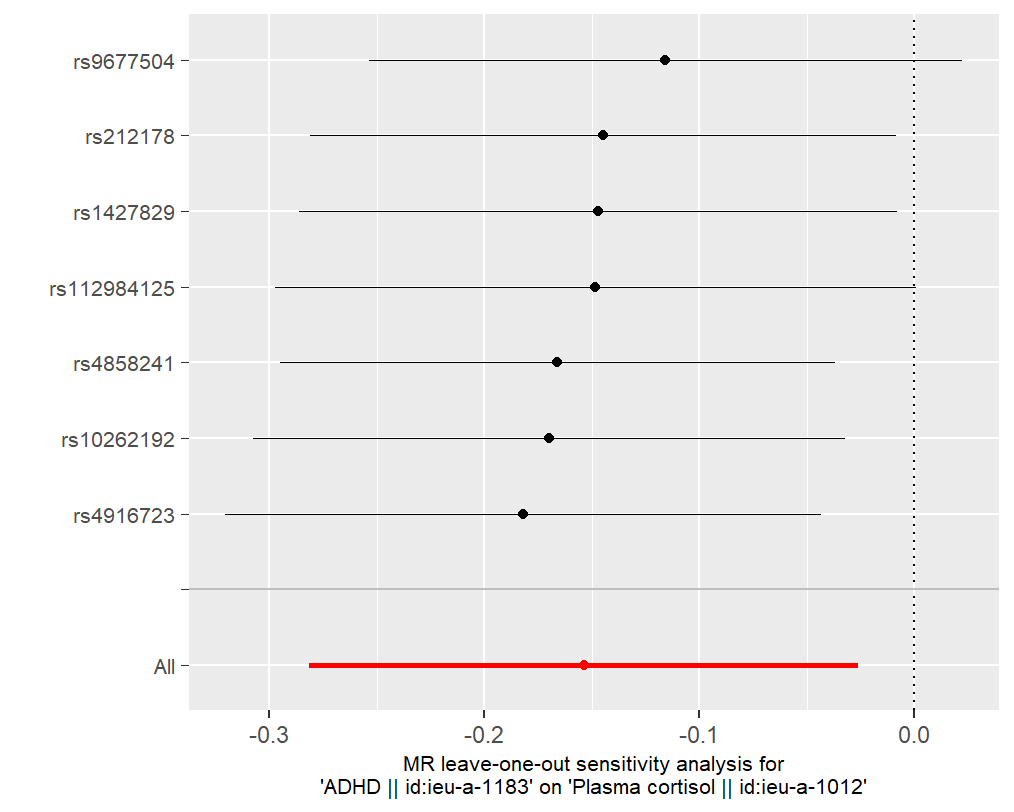
**

**MR leave-one-out sensitivity analysis for ‘ADHD’ on ‘Plasma cortisol’**
